# Carbon Nanotropes: A Contemporary Paradigm in Drug Delivery

**DOI:** 10.3390/ma8063068

**Published:** 2015-05-29

**Authors:** Avinash C. Tripathi, Shubhini A. Saraf, Shailendra K. Saraf

**Affiliations:** 1 Division of Pharmaceutical Chemistry, Faculty of Pharmacy, Babu Banarasi Das Northern India Institute of Technology, BBD City, Faizabad Road, Chinhut, Lucknow 226028, U.P., India; E-Mail: aviniec31@gmail.com; 2 Department of Pharmaceutical Sciences, Babasaheb Bhimrao Ambedkar University, Lucknow 226025, U.P., India; E-Mail: shubhini.saraf@gmail.com

**Keywords:** nanomaterials, carbon allotroptes, toxicity of carbon nanomaterials, CNTs, fullerenes, graphenes, drug delivery systems, anticancer drug delivery, delivery of biomoleculses, biosensors

## Abstract

Discovery of fullerenes and other nanosized carbon allotropes has opened a vast new field of possibilities in nanotechnology and has become one of the most promising research areas. Carbon nanomaterials have drawn interest as carriers of biologically pertinent molecules due to their distinctive physical, chemical and physiological properties. We have assigned the nomenclature “Carbon Nanotropes” to the nanosized carbon allotropes. Carbon nanotropes such as fullerenes, carbon nanotubes (CNTs) and graphenes, have exhibited wide applicability in drug delivery, owing to their small size and biological activity. The nanotherapeutics/diagnostics will allow a deeper understanding of human ills including cancer, neurodegenerative diseases, genetic disorders and various other complications. Recently, nanomaterials with multiple functions, such as drug carrier, MRI, optical imaging, photothermal therapy, *etc*., have become more and more popular in the domain of cancer and other areas of research. This review is an endeavor to bring together the usefulness of the carbon nanomaterials in the field of drug delivery. The last section of the review encompasses the recent patents granted on carbon nanotropes at United State Patent Trademark Office (USPTO) in the related field.

## 1. Introduction

The fullerenes, carbon nanotubes (CNTs), graphenes, sp-sp^2^ graphynes and many other synthetic allotropes are the latest addition to the family of the element carbon, in conjunction with the two kinds of graphite, two kinds of diamond, chaoit and carbon. These new allotropes of carbon are nanosized and, thus, we have coined the term “carbon nanotropes” for them. The nanotropes of carbon have opened a vast new field of possibilities in nanotechnology and have become one of the most promising research areas. Synthetic carbon allotropes represent a growing family of fascinating, and aesthetically pleasing, architectures with outstanding material properties.

In the family of synthetic carbon nanotropes, fullerenes correspond to the most rigorously explored class. Many well defined derivatives with exceptional properties have been synthesized under fullerene chemistry. Organic solar cells have already entered the market as the first fullerene-based product. The material properties of the CNTs, as well as especially those of graphene, are believed to be even more promising. Conversely, it is still not easy to control the chemistry and bulk production of the monodisperse samples. Graphene chemistry is in its premature infancy; however, there are a huge number of indefinable carbon allotropes whose predicted properties are unmatched. Synthetic chemists have already synthesized partial structures and are conceptualizing at present for their preparation [1].

## 2. Structure and Properties of Carbon Nanotropes

In 1985, a new allotrope of carbon (named Buckminsterfullerene) was discovered by Harold W. Kroto, Robert F. Curl and Richard E. Smalley for which they received the Noble Prize in the field of chemistry in 1996. The new form was found to consist of a truncated icosahedron, following the architect Buckminster Fuller who designed geodesic domes. The basis of closed cage icosahedra symmetry structure of fullerenes was 20 hexagonal and 12 pentagonal rings, in which every carbon atom was sp^2^ hybridized and bonded to three other carbons. There are two types of bond lengths found in a C_60_ molecule; 6:6 ring bonds can be deemed as “double bonds”, which are shorter than the 6:5 bonds. Similar to graphite structures, fullerenes are composed of linked hexagonal, pentagonal (sometimes heptagonal) rings of stacked graphene sheets to give potentially porous molecules. Fullerenes/carbon nanotubes are similar in their molecular framework, entirely composed of an extensive π-conjugated carbon skeleton. C_60_ Fullerenes avoid double bonds in the pentagonal rings, which results in poor electron delocalization and therefore does not behave as a “superaromatic” molecule. As a result, C_60_ fullerene behaves like an electron deficient alkene hence reacts eagerly with nucleophiles. The geodesic and electronic bonding factors are responsible for the stability of the molecule. Spherical fullerenes are called buckyballs, and cylindrical ones are known as carbon nanotubes or buckytubes. Buckyball clusters, more commonly known as endohedral fullerenes (consist of less than 300 carbon atoms) including C_60_ (buckminsterfullerene), are the most common froms of the fullerenes. Megatubes are prepared with walls of different thickness and larger in diameter than nanotubes, which are potentially used to carry a variety of molecules of different sizes. As an important member of the fullerene family, a buckyball core surrounded by multiple carbon layers growing into spherical particle is known as “nano-onion”, which endows it with some special properties.

**Figure 1:**
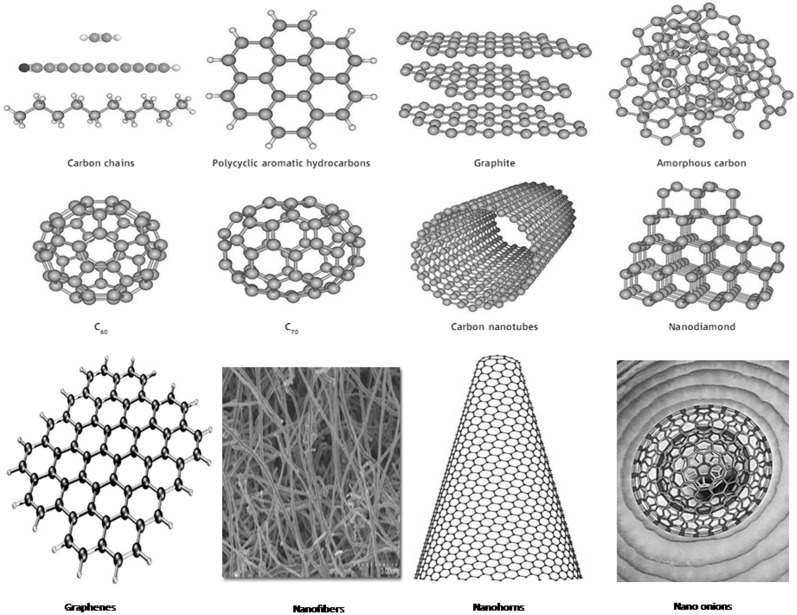
Different forms of carbon nanotropes [1].

CNTs are allotropes of carbon with a cylindrical nanostructure (Figure 1), discovered in 1991 by Sumio Iijima [2] of NEC. CNTs possess tensile strength hundred times more than that of steel, better thermal conductivity than diamond and electrical conductivity comparable to that of copper. On the other hand, these have the ability to carry much higher currents and hence seem to be a wonder material. Nanotubes exist in a variety of flavors such as; single-walled, multi-walled, long, short, open, closed, with different types of spiral structure and each type has specific production costs and applications. CNTs are classified into single-walled varieties (SWNTs), and multi-walled varieties (MWNTs). These are normally microscopic instead of nanoscopic, *i.e.*, greater than 100 nanometers and the lengths of both types vary to a great extent, depending on the way they are made. The aspect ratio (length/diameter) is usually greater than 100 and can be up to 10,000. In recent years, SWNT strands of 20 cm long (more recently 160 cm) were prepared, but the precise composition of these strands has not so far been made clear. SWNTs are the most important members of the nanotube world, and somewhat solitary ones at that, being much harder than the multi-walled variety to make them. SWNTs are more pliable than their multi-walled counterparts and can be flattened, twisted and bent into small circles. Because of their greater complexity and variety, the structures of multi-wall nanotubes are less implicit than SWNTs.

The single-walled carbon cones, produced by high temperature treatment of fullerene soot, having similar structures to those of nanotube caps are known as nanohorns. Nanohorns have good adsorptive and catalytic properties (substances stick to them and they enhance chemical reactions). Nanofibers are hollow and solid carbon fibers with lengths in the order of a few microns and width varying from some tens of nanometers to around 200 nanometers. These materials do not have the cylindrical chicken wire structure of SWNTs and MWNTs however, consist of a mixture of forms of carbon; graphite stacked at various angles to amorphous carbon (lacking any large-scale regular structure). Carbon nanobuds are a newly discovered allotrope of carbon in which fullerene like “buds” are covalently attached to the outer sidewalls of the CNTs. This hybrid material has the valuable properties of both fullerenes and carbon nanotubes. Because of their variable structure, these nanomaterials do not exhibit the strength of pure nanotubes but can still be quite strong and possess other useful properties.

The youngest synthetic carbon allotrope is a two-dimensional graphene (Figure 1), representing a single graphite sheet. Of the three families of carbon allotropes, graphene is the most structurally uniform material where only the sheet extensions and nature of edges can differ. Graphene, the ultimate example of expanded aromatic carbon, was considered for a very long time to be an exclusively theoretical material. Recently, however, single graphene layers were prepared successfully by means of a simple mechanical exfoliation of graphite using Scotch tape. Andre Geim and Konstantin Novoselov, were awarded the 2010 Nobel Prize in Physics, for the investigation of the electronic properties of grapheme [3]. Graphene produced an very high opacity in vacuum and appeared to be one of the strongest materials known with a breaking strength over 100 times greater than that of imaginary steel film of the same thickness [4,5]. Graphenes can exist in different forms such as; graphine oxide, chemically modified graphines, bilayer graphines, 3D graphines, *etc.* These nanomaterials can be useful in medicine, integrated circuits, transistors, redox, transparent conducting electrodes, ethanol distillation, desalination, solar cells, quantum dots, optical modulators, energy storage, biodevices, *etc.*

Buckyball soots are very finely divided black powder, while fullerites are brown/black powder and C_60_ are black solids. Fullerenes are virtually insoluble in acetone, ethers, and alcohols. C_60_ is essentially insoluble in polar solvents but sparingly soluble in alkanes. In aromatic solvents and in carbon disulfide, appreciable solubilities are observed. Some common physical properties of fullerenes are: density; 1.72 g/cc, melting point; 260 K, boiling point; sublimes at 800 K, standard heat of formation; 9.08 K·cal/mol, index of refraction; 2.2 (600 nm), resistivity; 1014 ohms/m, vapor pressure; 5 × 10^−6^ torr at room temperature [6,7,8,9,10,11].

Carbon nanotubes are the strongest and stiffest materials discovered till date in terms of tensile strength and elastic modulus. Covalent sp^2^ bonds between the individual carbon atoms are responsible for this strength [12]. Standard SWNTs can endure a pressure up to 24 GPa without showing deformation. MWCNTs exhibit a conspicuous telescoping property. However, an inner nanotube core may slide without friction within its outer nanotube shell, thus turning out to be an atomically ideal linear or rotational bearing. This is among the first true examples of molecular nanotechnology, which has already been utilized to produce the smallest rotational motor. The structure of a nanotube sturdily affects its electrical properties because of symmetrical and distinctive electronic structure of graphene, thus behaving like a moderate semiconductor [13]. There have been reports of intrinsic superconductivity in carbon nanotubes [14]. One of the more recently observed properties of multi-walled carbon nanotubes (MWNTs) is its wave absorption characteristics, specifically microwave absorption.CNTs are very good thermal conductors, exhibiting “ballistic conduction”, along axis, but excellent insulators laterally to the tube axis. SWNT has a thermal conductivity along its axis of about 3500 Wm^−1^K^−1^. The estimated temperature stability of carbon nanotubes was found to be 2800 °C in vacuum and about 750 °C in air [15].

Due to their unique properties, many potential applications, such as energy storage materials, high-performance and high-temperature wear-resistance materials, superconductive materials and biomaterials have been widely proposed for the carbon nanomaterials [16,17,18].

## 3. Cellular Uptake and Biodistribution of Carbon Nanotropes

Interactions of engineered nanomaterials (ENMs) with environmental interfaces have become a critical aspect of environmental health and safety evaluations. Carbon fullerene (C_60_) has emerged at the forefront of nanoscale research and applications due to its unique properties. Although there are concerns associated with the harmful effects of fullerene towards living organisms, the mechanisms of fullerene toxicity are still under debate. A first step toward assessing these mechanisms requires evaluation of the bio-accumulation and bio-uptake of fullerene through lipid membranes, which serve as biological barriers in cells. Evidences suggest that the lipid membrane composition can be a critical factor for assessing bioaccumulation of fullerene [19,20]. It is also important to understand the mechanism of engineered CNT based delivery systems to intracellular targets for the internalization of CNTs into the live cells, and has been considered to be decisive. The ability of CNTs to be internalized into a model lipid bilayer as a function of their length was explored by molecular dynamics (MD) simulations and statistical models, on highly functionalized and closed SWCNT, which revealed a large propensity of their passive uptake by phospholipid cell membrane. Hydrophobicity and membrane asymmetry are the key factors responsible for the insertion and complete translocation. Data demonstrates that shorter nanotubes have a stronger propensity to passively penetrate the bilayer and reach the cytoplasm [21]. Although much progress has been made in understanding how CNTs traverse the lipid membrane of a given cell type, the details of the proposed mechanisms are still debated. Such considerations are important in that the failure to understand the uptake mechanisms of nanoscale materials and their influence on toxicity could create another level of unpredictability. Internalisation to the cell has always been a preferred mechanism of drug delivery. It has been reported that functionalized MWCNTs are uptaken by cells in an energy independent manner. This holds true for various types of cell lines that undergo phagocytosis and also the ones that do not. Thus, the drug payload is effectively carried across the cell membrane and the same is effectively delivered, as well [22]. To date, two major mechanisms have been widely considered: (a) Endocytosis/phagocytosis and (b) Nanopenetration [23,24].

Endocytosis represents the engulfing of an extracellular particle by the cell, for example viruses (~100 nm in size), through the creation of a vesicle that is then integrated into the cell. Phagocytosis is similar to endocytosis but usually involves uptake of larger particles, such as bacteria (~1 μm), and is characteristic to a subset of immune cells/phagocytes (e.g., neutrophils, macrophages, dendritic cells). These processes are energy dependent and are hindered at low temperatures and in low ATP environments. Several studies implicate endocytosis/phagocytosis as the cellular uptake mechanism of CNTs [25].

Nanopenetration is an energy-independent passive process, where the nanotubes diffuse across the cellular membrane. In this respect, it is similar to passive diffusion of nano-needles from extracellular to intracellular space [26]. It was also pointed that CNTs could behave similarly to cell penetrating peptides (CPPs), which represent poly-cationic sequences that enhance uptake of proteins into mammalian cells. An interesting study in this context was the passive penetration of water-solubilized fluorescein isothiocyanate–attached CNTs and G-protein–functionalized CNTs into fibroblasts and keratinocytes at 37 °C. Such investigations hint that *f-*CNTs that resemble CPPs in morphology and possess an overall charge may more likely penetrate the plasma membrane rather than undergo endocytosis. Further experimental testing is crucial in that the type of functionalization could ultimately determine the precise mechanism. Despite of it, evaluation of the partitioning thermodynamics of fullerene demonstrated that the partitioning mechanism of fullerene is different from that of molecular level chemicals. It is generally acknowledged that molecular level chemicals partition into the hydrophobic center of lipid membranes (*i.e.*, absorption). However, the partitioning mechanism of fullerene is a combination of adsorption on the lipid membrane surface and absorption. Energy dependent endocytosis is hypothesized to be the main cellular transport mechanism based on an observed temperature dependence of cellular uptake and evidence for saturation of the active sites of transport during cellular uptake of fullerene. Metabolic inhibitors decrease the mass of fullerene taken up by the cells, which supports an active transport mechanism of fullerene through the cell membranes.

Different studies illustrated the significant contribution of plasma membrane translocation in the cellular uptake of CNTs. Multiple internalization pathways may simultaneously operate and determine CNT cellular uptake and trafficking. Furthermore, the balance between the possible mechanisms in operation will be strongly dependent on the type of CNT functionalization (*i.e.*, small organic groups or molecules against macromolecules or biopolymers like lipids, proteins or DNA), the physicochemical nature of the CNT dispersions (*i.e.*, individualization against small or large bundles) and the type of cells (*i.e.*, non-phagocytic against phagocytic cells). In general, it can be concluded that there was no single, unique mechanism responsible for CNT cellular uptake, and that chemical functionalization could represent a way to tailor the fate of CNTs by tilting the balance towards specific mechanisms of internalization, cellular processing and elimination/degradation depending on the desired application [22].

Mao *et al.* investigated the uptake, intracellular distribution and cellular effects of the collagen-SWCNTs, using them for culture of bovine articular chondrocytes (BACs). Well-dispersed SWCNTs in aqueous solutions can be obtained through functionalization of SWCNTs with type I collagen. The inherent properties of SWCNTs were retained after collagen functionalization and the collagen-SWCNT suspension was stable for over 63 days. The collagen-SWCNTs did not show any detrimental cellular effects on the BACs. Cellular uptake of collagen-SWCNTs by BACs was confirmed and up to ten million SWCNTs were internalized in one cell on an average. The distribution of collagen-SWCNTs in the cells was most prevalent in the perinuclear region. The results suggested that SWCNTs functionalized by collagen should be suitable for applications in biomedicine and biotechnology [27].

In comparison to large sized particles, nanoparticles show a different biodistribution profile, accumulation in tissues/organs, organ specificity, body clearance and chemical composition. These parameters play an important role in biodistribution studies and *in vivo* interactions of nanoparticles. Studies carried out so far point at involvement of physical clearance processes (viz., mucociliary movement, epithelial endocytosis, interstitial translocation, lymphatic drainage, blood circulation translocation and sensory neuron translocation) and chemical clearance processes such as dissolution, leaching and protein binding. Certain kinds of nanoparticles can pass through the GIT and are rapidly eliminated in feces and in urine, indicating the absorption across the GIT barrier and entry into the systemic circulation. However, some nanoparticulates can accumulate in the liver during first-pass metabolism. After intravenous administration, nanoparticles get distributed to the colon, lungs, bone marrow, liver, spleen, and the lymphatics. Such distribution is followed by rapid clearance from the systemic circulation, predominantly by action of the liver and spleenic macrophages clearance and opsonization of nanoparticles depends on size and surface characteristics. Differential opsonization translates into variations in clearance rates and macrophage sequestration of nanoparticles. To increase the passive retention of nanomaterials in systemic circulation, the suppression of opsonization events is necessary at desired sites or anatomical compartments. For example in case of hydrophobic particles, a coating with poly(ethylene) glycol (PEG), would increase their hydrophilicity, hence increasing the systemic circulation time [28].

The understanding of the mechanisms involved in the interaction of biological systems with nanomaterials is of interest to both, fundamental and applied disciplines. The adsorption of proteins modulates the formation of bio-films onto surfaces, a process important in infections associated with medical implants, in dental caries and in environmental technologies. A deep understanding of the mechanisms driving the interaction between biological fluids or cell constituents and surfaces is instrumental in designing strategies apt to prevent the toxicity and premature clearance of nanoparticles used in diagnosis and therapy, and for avoiding adverse reactions to materials used as implants or toxic effects that may follow the accidental exposure of organisms to nano-materials. The physico-chemical properties of the surface definitely play a pivotal role in modulating the various possible processes at the interface between biological fluids and solid surfaces; the peculiar features and behavior of bio-macromolecules further complicate the picture [29].

Quite a few studies are focused on the development of CNT and other carbon nanomaterial-based delivery systems. Attempts to systematically expound the mechanisms of cellular uptake of carbon nanotropes are still rather limited in the presence of different uptake inhibitors. Therapeutic or diagnostic cargos loaded into the nanocarbons showed the release of active molecules directly into the cytoplasm and increased their biological activity along with the therapeutic efficacy [22,30].

## 4. Toxicity of Carbon Nanotropes

In the last few years, both SWCNT and MWCNT have been utilized as nanocarriers for parenteral drug and gene delivery, and recently as targeted cancer treatment. The safety of CNT and other carbon nanotropes is still debatable due to the lack of systematic and complete toxicity evaluation. Some common cellular and tissue toxicity noticed with these carbon nanomaterials have been summarized in Table 1. The size of aggregated CNT is thought to be a primary concern for toxicity. Recently, studies have indicated that CNT responses are similar to the carcinogenic responses of asbestos fibers when injected into the peritoneal cavity [31]. Due to the high aspect ratio of CNT (>100), it is expected that CNT would behave as biopersistent fibers *in vivo*. Studies have implicated size (aggregation), CNT length, and manufacturing impurities as sources for potential toxicity *in vivo* [32].

**Table 1:** A compilation of cellular and tissue toxicity studies of pristine or functionalized carbon nanotropes [33].

Nanotube	Biological System	Dosage	Toxicity
Plasmid DNA-SWCNT and Plasmid DNA-MWCNT	f-CNTs: HeLa cell lines *in vitro*	10 mg/mL	50% survival of HeLa cells
Fluorescein isothiocyanate-SWCNT and fluorescein isothiocyanate-MWCNT	f-SWCNT and f-MWCNT: HeLa cell lines *in vitro*	5–10 mg/mL	50% survival of HeLa cells
Pristine SWCNT	SWCNT: Mesothelioma cell line MSTO-211H *in vitro*	7.5 μg/mL water	10% decrease in cell proliferation and activity
Ammonium chloride-SWCNT, and poly(ethylene glycol)-SWCNT	Macrophages, B and T lymphocytes from BALB/c mice spleen and lymph nodes *in vitro*	10 μg/mL water	5% decrease in viability of B lymphocytes, but no adverse effects on T lymphocytes and macrophages
RNA-polymer SWCNT conjugate	MCF-7 breast cancer cells *in vitro*	1 mg/mL	No significant cell damage
[^111^In] DTPA-SWCNT and [^111^In] DTPA-MWCNT	Intravenous injection, systemic, female BALB/c mice *in vivo*	20 μg/μL PBS	No acute toxicity after single 200 μL dose
Pristine MWCNT	Human T lymphocytes *in vitro*	40 μg/mL	Should have no toxicity on human T lymphocytes
Pristine SWCNT	Intravenous injection, systemic, rabbit *in vivo*	7.5 mL of 20 μg/kg body mass	No toxicity
^125^I-SWCNT (OH)	Intraperitoneal, intravenous, subcutaneous, in male KM mice *in vivo*	1.5 μg/mouse	Accumulate in bone, but good biocompatibility
Glucosamine-MWCNT	Intraperitoneally into female Kunming mice *in vivo*	300 μL single dose, suspension concentration unknown	Good biocompatibility
pEGFP-c1 plasmid DNA-SWCNT	Mouse B-cells and cortical neurons *in vitro*	0.1 pM/10 mL serum-free medium	~10% of cells were no longer viable
6-Aminohexanoic acid–derivatized SWCNT	Human epidermal keratinocytes (HEK) *in vitro*	Multiple tests from 0.00000005 to 0.05 mg/mL	Highest concentration that can interact with HEKs without toxicity, 0.000005 mg/mL for 24 h
DNA-Cy3 (fluorescent label)-SWCNT	HeLa cell line *in vitro*	2.5–5 mg/L water	No toxic effects, after six pulses of 10-s, 808-nm laser radiations at 1.4 W cm^2^
Streptavidin-SWCNT	HL60 and Jurkat cells *in vitro*	0.025 mg/mL	No adverse effects
SWCNTs dispersed in DMEM with 5% (vol/vol) fetal bovine serum	Human epithelial-like HeLa cells *in vitro*	100 μg/mL	No effect on growth rate

Pristine (non-functionalized) CNT are inherently hydrophobic; therefore, aggregation is expected and observed *in vivo*. For injection, pristine CNT are suspended in biocompatible surfactants such as Tween 80 or Pluronic F108. Several studies have been conducted on the *in vivo* distribution of intravenously injected pristine SWCNT. Primarily, accumulation of CNT was determined to be in the liver, but also in the spleen and lungs. No acute toxicity was observed in any tissue up to 24 h. Accumulation in the liver was suggested to be due to rapid surfactant displacement followed by opsonization of serum proteins. Distribution studies were followed up by looking at serum biomarkers of damage. Additionally, at markers for oxidative stress (glutathione and malondialdehyde) in liver and lung samples post dose, elevated levels of lactose dehydrogenase and alanine aminotransferase were concluded to be due to hepatic injury from accumulation in the liver. The study also found an increase in malondialdehyde and a decrease in glutathione in liver and lung samples (at 1.0 mg/mouse), which was indicative of increased levels of oxidative stress. Although no acute toxicity was determined histologically up to 90 days post dose, biomarkers indicating hepatic injury due to oxidative stress were further investigated [34]. Studies conducted longer than 90 days may exhibit more toxicity damage over time, similar to onset of damage from asbestos fibers in lungs, which could take years. ROS formation and cytotoxic effects induced by aggregates and accumulation have been observed in other studies of CNT [23]. Reduction of CNT accumulation and aggregation is achieved by functionalization [35]. Rapid distribution and renal clearance from almost all tissues was observed 1–3 days after injection of functionalized CNT, whereas pristine CNT were determined at high levels in RES tissues up to 1 month after injection. No comprehensive examination of functionalized CNT degradation-induced toxicity has been reported, but these are important questions as functionalized CNT have more promise as therapeutic and diagnostic tools.

Along with CNT aggregation as a function of improper macrophage uptake, the length of CNT has been implicated as a source of improper macrophage translocation. Some studies focused on the effect of length on CNT toxicity by injecting MWCNT i.p. and observing carcinogenic mechanisms in the abdominal cavity and on the diaphragm. The longer length (= 20 µm) CNT resulted in an inflammatory response within 24 h, with consequent granuloma 7 days after injection. These longer lengths may cause carcinogenic effects such as mesotheliomas, if longer studies were conducted. In the same study, shorter lengths of i.p injected MWCNT were effectively taken up by macrophages with efficient phagocytosis. In terms of CNT for therapeutics, it can be deduced that SWCNT may be more favorable than MWCNT from a toxicology standpoint due to smaller size and length resulting in less aggregation and better uptake by macrophages. It has been further suggested that MWCNTs were recognized and interacted with macrophage receptors on the plasma membrane and can rupture the membrane causing cytotoxicity and damage to the macrophage [36].

Methods of CNT fabrication and purification have been shown to increase toxic effects of CNT. Arc-discharge, laser ablation, chemical vapor deposition, and high-pressure carbon monoxide synthesis are methods commonly used to fabricate CNT. These methods are often performed in the presence of metal catalysts with CNT growth occurring on solid supports. Impurities such as residual metal or supports and amorphous carbon in the final formulation have been thought to induce oxidative stress. On the other hand, extensive purification and treatment will also cause degradation of the nanotubes [37]. Therefore, a fine balance between synthetic methods and purification must be achieved to fabricate highly purified CNT for injection. Low clearance and accumulation of CNT *in vivo* warrants studies to determine chronic exposure toxicity. Radiolabeled isotopes were commonly used to evaluate the *in vivo* performance of CNT. However, due to isotopic decay and degradation of the label from the CNT, radiolabeling suffers from short study timeframes. Therefore, estimation and evaluation of chronic exposure toxicity may be possible based on evaluation of CNT intrinsic properties.

Identification of pharmacological and toxicological profiles is of critical importance for the use of nanoparticles as drug carriers in nanomedicine and for the biosafety evaluation of environmental nanoparticles in nanotoxicology. Lysosomes have been considered as the pharmacological target organelles for single-walled carbon nanotubes (SWCNTs) and mitochondria as the target organelles for their cytotoxicity. The gastrointestinally absorbed SWCNTs were lysosomotropic, but also entered mitochondria at large doses. Genes encoding phosphoinositide-3-kinase and lysosomal-associated membrane protein 2 were found to be involved involved in such an organelle preference. SWCNT administration resulted in collapse of mitochondrial membrane potentials, giving rise to overproduction of reactive oxygen species leading to damage of mitochondria, which was followed by lysosomal and cellular injury [38].

Owing to their special properties, nanoparticles have the capacity to bypass the blood–brain barrier (BBB). The toxic effects of nanoparticles on central nervous system (CNS) function are still lacking, and their interactions with the cells and tissues in CNS are poorly understood. However, due to the large surface area, some of the nanoparticles may cause neurotoxicity after entering into the brain. Using a fish model, Oberdorster (2004) investigated the toxicity of fullerenes NPs on the brain of bass through the evaluation of oxyradical-induced lipid and protein damage as well as total glutathione (GSH) levels. Significant lipid peroxidation was found in the brains of largemouth bass after 48 h of exposure to 0.5 ppm uncoated C_60_ (30–100 nm). Therefore, the fullerene NPs could cause cell damage in the brains of fish [39].

In cases where CNTs have a toxic interaction with cells, the mechanisms of toxicity are coming into focus. Results suggest CNTs may cause harm to cells by activating many pathways at once, mostly involving DNA damage [40]. In one study, mesothelial cells exposed to SWCNTs, at concentrations ~25 μg/cm^2^ activated DNA recovery along with changes in the cell cycle and generation of apoptotic signals. Another approach showed that most cells incubated with CNTs halt at the G1 phase of the cell cycle [41]. It was also observed that CNT/DNA interaction was the preferred route of toxicity in a 3 h incubation study with 96 μg SWCNT/cm^2^, which induced DNA damage (through micronucleus generation) in lung fibroblasts [42]. It should be possible, through the observation of specific toxic events, that result from incubations with different types of f-CNTs, to test for functional groups that reduce the severity of such events.

In conclusion, toxicity concerns of general CNT use *in vivo* have stemmed from observed toxicity after delivery to the lungs. Current studies have shown that once in the bloodstream, intrinsic properties, propensity to aggregate and slow clearance can lead to oxidative stress especially in the liver, lungs and spleen, ultimately resulting in inflammation. More importantly, the length of CNT has been shown to result in inefficient phagocytosis and damage to macrophages. Due to less aggregation and shorter lengths, SWCNT are better suited for *in vivo* applications over MWCNT. Functionalization of SWCNT further reduces aggregation; however, more thorough research on functionalized CNT toxicity is required. Moreover, methodically conducted experiments that mimic chronic exposure to CNT will be more productive in the determination of CNT nanotoxicity [43].

## 5. Carbon Nanotropes in Drug Delivery

Nanoparticles are useful to control and manipulate bio-macromolecular constructs and supra-molecular assemblies that are critical to living cells. The nanoconstructs and assemblies including; drugs, proteins, DNA/RNA, viruses, cellular lipid bilayers, cellular receptor sites and antibody variable regions (decisive for immunology) are involved in events of nanoscale proportions. The nanotherapeutics/diagnostics may lead to deeper understanding of human ills such as cancer, cardiovascular disease and genetic disorders. Size, shape, surface chemistry and other nanotechnical dimensions are decisive steps in understanding scientific basis of nanoparticles [44]. Carbon nanomaterials such as fullerenes, CNTs and graphenes, *etc.*, show wide applicability in drug delivery, due to their small size and biological activity. Nanomolecular carbon cages like fullerenes are used for the delivery of drugs and imaging agents, in several functional modes. These are also useful drug vectors or drug delivery scaffolds with non-covalent or covalent linkages between the fullerene and a bioactive moiety [45]. Fullerene other carbon nanotroes have numerous points of attachments, which allowaccurate grafting of active chemical groups in 3D orientations. This attribute is the hallmark of rational drug design, which allows positional control in matching these carbon nanocompounds to biological targets. It is possible to modify some essential pharmacokinetic characteristics of carbon nanotropes and to optimize their therapeutic effect, in association with other attributes such as, size, redox potential and the relative inertness in biological systems [46]. Because of these peculiar characteristics, fullerenes hold great promise for biomedical applications [44,47,48,49,50]. The biological activities of carbon nanotropes depend upon the core and its chemical modification. Functional groups attached to the core put in more complexity to the behavior of the highly hydrophobic fullerene molecule. Hydrophilic functionalization results into water-soluble fullerenes, which is competent to carry drugs and genes for the cellular delivery. Derivatized fullerene binds to the mitochondria by crossing the cell membrane. Moreover, DNA-functionalized fullerenes are capable of entering into the COS-1 cells (fibroblast-like cell line derived from monkey kidney tissue) and offer better efficiency than that of commercial lipid-based vectors. A protective sheath of fullerene reagent is formed around the bound DNA. This increases the lifetime of DNA in endosomes and hence supports their chromosomal incorporation. Amino fullerenes are among the preferred nanomaterials for the attachment of DNA-sequences. Detachment of DNA in the cytoplasm is accomplished by loss of the amino groups or loss of the binding ability of amines by transforming them into neutral compounds [51,52]. In tissue culture, fullerene derivatives were employed to enhance the therapeutic efficacy of a lipophilic slow-release drug delivery system [53]. In C_60_-paclitaxel conjugate, modified fullerenes provide a lipophilic slow-release system exibiting significant anticancer activity in the cell culture. Furthermore, the ability of fullerene derivatives in penetrating through intact skin has broadened their application in cellular drug and gene delivery [54]. A fullerene-based peptide derivative has been synthesized and evaluated for its ability to penetrate through flexed and inflexed skin by Rouse *et al*. [55].

Development of safe and efficient carriers to transport genes or drugs has attracted interest in the area of direct delivery of drugs and biomolecules into cells through the cell membrane. Cell membrane, the endosomal membrane and the nuclear membrane barriers pose major challenges in transporting any compound into the nucleus of an intact cell. Therefore, it is important to understand the mechanism through which carriers enter the cells. Presently, organic cationic compounds, viral carriers, recombinant proteins and inorganic nanoparticles are the four major categories of drug and gene carriers. Because of good biocompatibility, selective targeted delivery and controlled release of carried drugs, a huge number of carbon nanotropes are being used in cellular delivery [56,57].

### 5.1. Delivery of Anticancer Drugs

Drug delivery systems are useful in handling the problems associated with the administration of anticancer drugs, such as poor solubility, limited biodistribution, nonselectivity, and damage of healthy tissues. A good range of materials such as; microspheres, liposomes, polymers and recently, CNTs, are available, which have been explored for delivering anticancer drugs. These substances are valuable in reducing the number of required administrations and increasing patient compliance, by providing more controlled and better use of the active agents. In recent times, nanomaterials with multiple functions have become more and more popular in the domain of cancer research, such as drug carrier, MRI, optical imaging, photothermal therapy, *etc.* Carbon nanotropes have drawn considerable attention as carriers of biologically pertinent molecules (Table 2) due to their distinctive physical, chemical and physiological properties. A precise association between the physical-chemical properties of carbon nanotropes, their reactivity, cell to-cell interactions, and systemic consequences are some important concerns and it is imperative to know such inter-relationships beforehand to enjoy the advantages of such nanomaterials without any hazardous consequences [58]. Carbon allotropes such as fullerenes, CNTs, graphene oxide (GOs) and nanodiamonds (NDs), have been investigated as excellent nanomaterials due to their adequate thermal conductivity, surface-to-volume ratio, rigid structural properties required for post-chemical modifications, and admirable biocompatibility [59]. The use of CNTs as multivalent tools for cancer treatment is making headway at a very fast pace. Targeted delivery of drugs, intended to selectively steer the therapeutic treatment towards the tumours, is coming out as the most promising approach. The achievements obtained so far in the field of drug delivery includes chiefly; anticancer chemotherapeutics (doxorubicin, methotrexate, taxanes, platinum analogues, camptothecin and gemcitabine), immunotherapeutics and nucleic acids. Moreover, the alternative anticancer therapies based on thermal ablation and radiotherapy have also been discussed in the past [60]. Because of nanoscale dimensions, high aspect ratio, enhanced drug loading efficiency, competency in penetrating mammalian cell membranes, SWCNTs have been recognized as anti-cancer drug transporters. In addition, these nanomaterials can assist the targeting of therapeutic agents to the desired site of action by conjugation with antibodies or ligands of cancer cell surface receptors, which increases the effectiveness of the treatment and reduces side effects [61].

Functionalized carbon nanotubes (*f-*CNTs) are promising nanomaterials for the development of unique delivery systems of anticancer drugs as demonstrated by Lay *et al*. Functionalization of CNTs and drug loading can be achieved by covalent attachment and/or by physical approaches. Poly(ethylene glycol) can increase the dispersity in aqueous solution and biocompatibility of CNTs, and hence is recognized as one of the most accepted agent for functionalization. Treatment efficiency of numerous anticancer drugs, such as paclitaxel and doxorubicin, has been demonstrated *in vitro* and *in vivo* by loading them with *f-*CNTs [62].

**Table 2:** Delivery of anticancer drugs through carbon nanotropes [63].

Drug delivery system	Dosage and biological system employed	Application	Method of drug release	Remarks
HCPT-diamino-triethylene glycol-MWCNTs [64]	5 mg kg^−1^ HCPT (Hepatic H22 tumor-bearing mice)	Gastric carcinoma	pH Triggered drug release	More efficient
C_60_-IONP-PEG/ Hematoporphyrin monomethyl ether (HMME) [65]	HMME, a new photodynamic anti-cancer drug, was conjugated to C_60_-IONP-PEG, forming a C_60_-IONP-PEG/HMME drug delivery system	Cancer theranostic	N.M.	Remarkably enhanced photodynamic cancer cell killing effect
Multi-functional C_60_-IONP-PEG-Folic acid (FA) [66]	Folic acid linked to C_60_-IONP-PEG in order to obtain an active tumor targeting effect to MCF-7 cells and malignant tumor in mice models	Cancer diagnosis, PDT, RF RTT and magnetic targeting	N.M.	Excellent physiological stability, neglegible toxicity, selectivity
Paclitaxel-ultrathin PLGA film-QD-MWCNT [67]	100 ng mL^−1^ Paclitaxel-PLGA-CNT (Nu/nu nude mice; 6–8 weeks old, about 18 g)	Prostate carcinoma	N.M.	More efficient in tumor treating and low toxic to living mice
Doxorubicin-FA-CHI/ALG-SWCNT [68]	50 μg mL^−1^ DOX-FA-CHI/ALG- SWCNT (HeLa cells)	Cervical carcinoma	FA–FA Receptor interaction, pH triggered drug release	More cytotoxic and selective
Doxorubicin-pluronic F127-MWCNT [69]	10 μg mL^−1^ DOX:20 μg mL^−1^ CNT (MCF-7 cells)	Breast cancer	N.M.	More efficient
Doxorubicin-amphiphilic polymers-CNT [70]	0.5 mg mL^−1^ DOX-CNT (B16F10 cells)	Melanoma	N.M.	More efficient
Doxorubicin/Folic acid-MWCNT@Fe [71]	32 μg DOX per mg of FA-MWCNT@Fe (HeLa cells)	N.M.	FA–FA Receptor interaction (active) and magnetic force (passive)	Prolonged drug release
{Pt(IV)}-PL-PEG-SWCNT [72]	65 Pint(IV) centers per nanotube (average), (NTera-2 cells)	Testicular cancer	pH Triggered drug release	Higher toxic to tumor cells
Electroactive polyimide-MWCNT [73]	131.3–120.8 mg EPI per gram of c-MWCNT	N.M.	N.M.	Greater EPI release in acidic medium
Doxorubicin/PEGylated MWCNT [37]	N.M. (Hela, HepG2, K562 cells)	Liver cancer and leukemia	N.M.	Efficient anti-multi drug resistance effect
Cis-Diammine dichloroplatinum–SWCNT [74]	100 μg mL^−1^ CDDP-CNT (DU145 and PC3 cells)	Prostate cancer	CDDP-Polynucleotide chain interaction	Similar effect on PC3 cells but less on DU145 cells
EGF-Cis-diammin dichloroplatinum–SWCNT [75]	1.3 μM CDDP in EGF-CDDP-SWCNT (Female athymic (nu/nu) nude mice (4–6 weeks old, weighing 18–20 g)	Squamous carcinoma	EGF–EGF Receptor interaction	More efficient
Conducting Polymer-MWCNT [76]	0.5 μg μL^−1^ CP-CNT (EJ28 cell line)	Bladder cancer	N.M.	N.M.
Biotin-SWCNT-cleavable disulfide linker-(taxoid-fluorescein) [77]	13.9 μM Taxoid (L1210FR, L1210 and WI38 cell lines)	Leukemia	Biotin-biotin receptors mediated endocytosis	More efficient
C_60_-HMM-SWCNT/DWCNT [78]	N.M.	N.M.	CH_2_Cl_2_Triggered drugs removal	N.M.
Oxaliplatin/MMC-MWCNT [79]	(300 μM oxaliplatin + 100 μg CNT) per mL medium (RKO and HCT 116 cell lines)	Colorectal cancer	IR radiation stimulated, hyperthermic method	N.M.
Methotrexateand 1,3-dipolar cycloaddition on MWCNT [80]	5 μg mL^−1^ Conjugate (Jurkat cells)	Jurkat cells	N.M.	N.M.
Methotrexate -Gly-Leu-Phe-Gly/6-hydroxy hexanoic ester on 1,3-dipolar cycloaddition *f*-MWCNT [81]	10mM MTX–MWCNT (MCF-7 cells)	Human breast carcinoma	N.M.	Higher cytotoxicity in MTX–MWCNT using peptide linker

Note: N.M.: Not mentioned.

Shi *et al*. synthesized a C_60_-IONP nanocomposite via integrating iron oxide nanoparticles (IONP) with fullerene (C_60_). The functionalization was done by polyethylene glycol (PEG2000), giving C_60_-IONP-PEG which showed excellent stability in physiological solutions. Finally, folic acid (FA), the most commonly used tumor targeting molecule was attached to C_60_-IONP-PEG in order to attain an active tumor targeting effect to MCF-7 cells and malignant tumors in mice models. This hybrid C_60_-IONP-PEG-FA nanoplatform with multi-functional characteristics for cancer diagnosis, photodynamic therapy (PDT), radiofrequency thermal therapy (RTT) and magnetic targeting applications was developed and explored for its biofunctions *in vitro* and *in vivo*. Moreover, this nanotechnology was used to selectively kill cancer cells in highly localized regions, and thus proves potential expediency of C_60_-IONP-PEG-FA nanoplatform in cancer theranostic applications [66].

Yang *et al.* demonstrated that graphene and its derivatives such as graphene oxide (GO), reduced graphene oxide (RGO) and GO-nanocomposites have recieved remarkable attention in the field of biomedicine, due to their peerless physical and chemical properties. Single-layered graphenes show ultra-high surface area available for efficient molecular loading and bioconjugation, and hence, widely used as novel nano-carriers for drug and gene delivery. *In vivo* graphene-based photothermal therapy was found to bring about excellent anti-tumor therapeutic efficacy in animal experiments, employing the intrinsic near-infrared (NIR) optical absorbance. Inorganic nanoparticles, grown on the surface of nano-graphenes, resulted into the formation of functional graphene-based nanocomposites with interesting optical and magnetic properties which were found to be useful for multi-modal imaging and imaging-guided cancer therapy. Also, a noteworthy effort was made to study the behavior and toxicology of functionalized nano-graphenes in animals [82]. Shi *et al.* brought together graphene oxide (GO) with iron oxide nanoparticles (IONPs) and gold (Au), to form a multi-functional magnetic and plasmonic GO-IONP-Au nanocomposite with strong superparamagnetism and enhanced optical absorbance in the near-infrared (NIR) region. Finally, the nanocomposite was coated with polyethylene glycol (PEG) to obtain GO-IONP-Au-PEG, with high stability in physiological environments and insignificant *in vitro* toxicity. Remarkably enhanced photothermal cancer ablation effect, using GO-IONP-Au-PEG, was observed in comparison to PEGylated GO. Results of this study pledge graphene-based multi-functional nanocomposites as cancer theranostics [83].

### 5.2. Delivery of Neurological Drugs

The use of targeted nanotherapies to cross the blod brain barrier (BBB), bind and act only on the target has made a great deal of difference in many diseases, particularly in CNS diseases where the flexibility of nanomaterials shows the greatest promise. Getting therapeutics to cross the BBB used to be an insurmountable barrier. Agents that were originally intended for the brain, based on their success in treating other diseases, ran into a major stumbling block when administered in the brain [84]. Cabron nanomaterial-based technology has been investigated as an alternative for the treatment of neurodegenerative diseases [85].

Only a few studies have so far been published on the use of carbon nanomaterials for brain drug delivery; however, this is likely to increase in the coming years. The emergence of CNT as a delivery vector for the CNS is based on their structural advantages, in particular good dispersibility in physiological solvents, large surface area, capability of being easily functionalized with drugs or imaging agents and biocompatibility with neural tissue [86]. Alzheimer’s disease (AD) is presently affecting more than 35 million people and represents the most common form of dementia worldwide. Advances in nanotechnology have promise to exert a considerable impact in neurology. Nanoparticles (NPs) showing high affinity for the circulating amyloid-*β* (A*β*) forms were found to induce “sink effect” and improve the AD condition. There are also developments in relation to *in vitro* diagnostics for AD, including ultrasensitive NP-based bio-barcodes, immunosensors, as well as scanning tunneling microscopy procedures capable of detecting A*β*_1−40_ and A*β*_1−42_ [84]. For the first time, Zhang *et al.* used CNT for the treatment of CNS diseases. They have utilized short pristine SWNT physically adsorbed with acetylcholine (SWNT-ACh) in Alzheimer’s disease brains. Because SWCNTs have the ability to absorb inorganic and organic chemicals, in addition to the ability to enter the brain via nerve axons, they are ideal for the delivery of ACh molecules. This study suggested that doses of SWNT under 300 mg/kg, after gastrogavage administration, could ensure safe delivery of ACh into lysosomes of neurons, thus rendering the therapy more effective without compromising the toxicological profile of the material. Once SWNT-ACh enters inside the brain lysosomes, increased polarity and hydrophilicity of ACh at the low lysosomal pH, brings ACh into play as a neurotransmitter. There are different target organelles responsible for the pharmacological and toxicological effects of SWCNTs. The lysosomes are the pharmacological target organelles, whereas the mitochondria are the target organelles of SWCNT toxicity. All nanomaterials may be toxic in a strict sense, but their toxicity does not necessarily militate against their application in nanomedicine. The key is to control the dosage. At high doses SWCNTs did cause pathological changes in the ultra-structures of lysosomes and mitochondria; they are highly safe at low doses, providing the basis for their use as drug carriers [38].

Another possible therapeutic application of CNT in the CNS is in the treatment of glioblastoma tumours, which are known for their recurrence even after aggressive multimodality treatment. The treatment of brain tumours remains a challenge despite advances in tumour therapy and the increasing understanding of carcinogenesis. The low permeability of anti-tumour drugs across the BBB, when administered systemically, has opened up new possibilities for CNT-based modalities. For example, Zhao *et al*. [87] have recently demonstrated that the CNT delivery system significantly enhanced CpG oligodeoxynucleotides immunotherapy; eradicating the glioma and protecting against tumour rechallenge (GL261 and GL261egfp models).

CNT-mediated therapy is a valuable option for the treatment of neurodegenerative diseases, including the treatment of stroke. In this context, the potential use of amine-functionalized SWNT was evaluated by Lee *et al.* [88], using a middle cerebral artery occlusion (MCAO) stroke model, to enhance the survival of neurons following ischemic injury. It was observed that the intracerebroventricular injections of SWNT without any therapeutic molecule enhanced the motor function recovery of the animals; however, the mechanism for such activity remains elusive. Recently, in vivo effectiveness of amino-functionalized MWNT was also demonstrated in the delivery of siRNA (specific to silence caspase 3), promoting behavioral recovery in endothelin-1 stroke murine models. Unlike the study by Lee *et al.*, no neuroprotective activity was attributable either to CNT alone or CNT complexed with scrambled siRNA. The GL261 murine intracranial glioma model was used to study the uptake and toxicity of MWCNTs and to evaluate the potential application of CNTs in brain tumor therapy. Nearly 10%–20% of total cells demonstrated CNT internalization within 24 h of a single intratumoral injection of labeled MWCNTs (5 µg). About 75% CNT uptake was observed in tumor-associated macrophages (MP), which accounted for most MWCNT-positive cells. Nearly 30% of tumor MP became MWCNT-positive within 24 h of injection. No considerable toxicity was noticed in mice and only minor changes in tumor cytokine expression were observed, in spite of a transient increase in inflammatory cell infiltration into both normal and tumor-bearing brains following MWCNT injection. This study suggested that MWCNTs could be used as potentially novel and non-toxic vehicles to target MP in brain tumors [89].

### 5.3. Delivery of Anti-Tubercular Drugs

The potential application of CNTs pertinent to drug delivery is highly apparent, due to its notable electronic and structural properties. Density functional theory (DFT) calculations were performed by Saikia *et al.* to simulate the interaction of nanomaterials with biomolecular systems, by studying interaction of pyrazinamide (PZA) drug with *f-*SWCNT as a function of chirality and length. Two different approaches of covalent functionalization were used, followed by docking simulation of *f-*SWCNT with pncA protein. The formation of nanotube-drug conjugate is thermodynamically feasible and the pristine SWCNT functionalization facilitates in enhancing the reactivity of the nanotubes. Docking studies predicted the probable binding mechanism and suggested that PZA loaded *f-*SWCNT facilitated the target specific binding of PZA within the protein through lock and key mechanism. A hydrophobic interaction was observed between ligand and receptor, and more interestingly it previewed, that there was no structural deformation in protein structure on binding with CNT. These findings spotlighted some new drug delivery mechanisms by CNTs with long term practical implications in tuberculosis chemotherapy [90].

In another study, Gallo *et al.* demonstrated the utility of DFT calculations to scrutinize the effects of covalently binding isoniazid, an anti-tubercular compound, to *f-*CNTs and *f-*C_60_s. Binding energies, energies of solvation, quantum-chemical molecular descriptors were calculated. Results from binding energies indicated that *f-*C_60_s bound isoniazid more easily than the *f-*CNTs. Solvation energy calculations showed that the solubility of *f-*CNTs in water was higher than *f-*C_60_s, and was thermodynamically favorable. It was also concluded from the studies that increasing isoniazid substitution in functionalized fullerenes resulted in a decrease in global hardness, which in turns decreased their stability [91].

Dapsone (dap), an anti-microbial and anti-inflammatory drug, was modified onto *f-*MWCNTs by Vukovic *et al*. Non-obvious apoptosis of rat peritoneal macrophages were observed when, dap-CNTs or oxidized CNTs (o-CNTs), up to 50 μg mL^−1^, were used. Higher levels of both types of CNTs induce apoptosis, which was greater in the case of o-CNTs. Furthermore, prolonged incubation of cells (>3 days) in 50 μg mL^−1^ of dap-CNTs also triggered apoptosis. Similar levels of individual dapsone and o-CNTs caused oxidative stress, whereas dap-CNTs did not cause any oxidative stress. Therefore, dap-CNTs could be effectively used for treating dap-sensitive intracellular microorganisms and dap-responsive inflammatory diseases [92].

### 5.4. Delivery of Anti-Fungal Drugs

Amphotericin B (AmB) is a frequently used intravenous antifungal drug of polyene category for the treatment of systemic fungal infections. This drug was found to be associated with some serious and potentially lethal side effects to mammalian cells, including acute infusional reactions and a dose-dependent nephrotoxicity, due to its poor water solubility, resulting in aggregates causing this toxicity [93]. It could be minimized by increasing the water solubility through binding of AmB to *f*-CNT. Drug efficacy will be improved and the antibiotic activity can be modulated through binding to carbon nanotropes. *f*-MWCNTs were used for the targeted delivery of AmB. MWCNTs were treated with acid to introduce the carboxylic groups, followed by functionalization with two orthogonally-protected amino acids and finally, conjugated with fluorescein isothiocyanate (FITC). High antifungal activity of AmB was preserved even after binding to MWCNT. The AmB-CNT complex was found to be transported across the mammalian cells without showing any symptom of cytotoxicity [94]. Furthermore, micellar dispersion of AmB with sodium deoxycholate (AmBD) has been reformulated by Benincasa *et al.* [95], and finally encapsulated into liposomes by incorporating into lipidic complexes. Two conjugates of *f*-CNTs and AMB were prepared and tested for their antifungal activity against various fungal strains, when compared to that of AmB alone or AmBD. Measured minimum inhibitory concentration (MIC) values of *f*-CNT-AmB conjugates were found comparable to or even better than, that displayed by AMB and AmBD. In addition, AmBD-resistant *Candida* strains were also found to be susceptible towards *f*-CNT-AmB1. A nonlytic mechanism was suggested for the action of these conjugates since after extended incubation, the compounds showed foremost permeabilizing effect on the fungal strains only. Interestingly, the *f-*CNT-AmB1 at antifungal concentrations was not found to show any notable toxic effect on Jurkat cells.

### 5.5 Delivery of Anti-Inflammatory Drugs

Dexamethasone (DEX) is one of the most commonly used immunosuppressant and anti-inflammatory drug to treat many autoimmune and inflammatory diseases. SWCNTs have been used as host-carrier film for the electrically stimulated delivery of DEX. An accelerated cellular uptake and a complete drug release of DEX was observed due to electrostatic repulsions between SWCNTs and DEX when −0.8 V potential was applied. The passive release of DEX was decreased by the addition of SWCNTs, due to the possible attractive interactions between the drug and SWCNTs [96].

Ketoprofen, a widely used non-steroidal anti-inflammatory drug with analgesic and antipyretic effects, inhibits the production of prostaglandin in the body, is commonly prescribed for the treatment of inflammatory conditions due to arthritis or severe toothaches caused by gum inflammation. An electro-sensitive transdermal DDS, composed of a semi-interpenetrating polymer network (polyethylene oxide-pentaerythritol triacrylate) as the matrix and MWCNTs was demonstrated to increase the electrical sensitivity of (S)-(+)-ketoprofen. The amount of released drug increases with enhanced applied potentials, which can be attributed to higher electrical conductivity of CNTs [97].

### 5.6. Delivery of Topical Agents

A great deal of attention has been focused on determining the properties of nanoparticles that might serve as efficient drug delivery matrices [98]. Chemically functionalized, water-soluble SWNTs were found to enter fibroblasts [99], promyelocytic leukemia (HL60) cells and T cells [100]. However, little attention has been paid to the ability of unmodified nanotubes, manufactured for engineering applications, to localize within cells. Evidence of dermal irritation, coupled with a report of toxicity to keratinocytes, suggests that particles not optimized for intracellular delivery may enter cells and adversely affect cellular function. Human epidermal keratinocytes (HEK) were exposed to 0.1, 0.2, and 0.4 mg/mL of MWCNTs for 1, 2, 4, 8, 12, 24 and 48 h and were examined, by transmission electron microscopy, for the presence of MWCNT. Chemically unmodified MWCNT were found to be present within cytoplasmic vacuoles of the HEK in all time frames. The MWCNT were found to induce the release of the pro-inflammatory cytokine interleukin 8 from HEKs with time frames. These facts evidently explained that non derivatized MWCNT were capable of both, localizing within and initiating an irritable response in a target epithelial cell which comprise of a primary route of occupational exposure for manufactured nanotubes. Transmission electron microscopy (TEM) confirmed that for MWCNT neither chemical modification nor other substances (vehicles, surfactants, and other solubilization strategies) were helpful in entering HEK. An increase in the release of IL-8 from the treated cells confirmed that a biological response had occurred. However, this response may be the cumulative effect of both, MWCNT attaching to the plasma membrane as well as being internalized by the cell. This marker of irritation (IL-8) is consistent with reports of dermal irritation in humans. These studies were not designed to develop a precise dose–response relationship for nanotube exposure, nor define the relation of nanotube properties (surface properties, size, *etc.*) to cellular penetration. It was obvious in exposures that the vast majority of nanotubes dosed in the media did not interact with keratinocytes. As was typically seen with these structures, clumping and aggregation occurred during exposure. However, after filtering out large aggregates, nanotubes were still present within the keratinocytes. The importance of these findings is that detectable fractions of chemically unmodified MWCNT were capable of intracellular localization as well as causing irritation in keratinocytes by IL-8 release. In short, keratinocytes can be affected by MWCNT [101].

### 5.7. Delivery of Biomolecules, Gene Transfection and As Biosensors

In addition to drugs, CNTs have also proven their utility in the delivery of biomolecules (Table 3). Proteins bind covalently to SWCNTs/MWCNTs by diimide-activated amidation to form CNT-protein conjugates with high water solubility [102]. The delivery of functional genes to target cells for achieving therapeutic effect is defined as gene therapy. Gene therapy is being considered as a potential medical revolution. Initially, it was viewed as an approach for treating hereditary diseases, but now wide recognition of its potential role in the treatment of acquired diseases such as cancer is being envisaged. DNA can also be attached to the amino groups of *f*-MWCNT. The linkage of DNA to *f*-MWCNT is utilized to improve the dispersibility of nanotubes in aqueous media as well as for competent gene transfection, without using viral genes [103]. The need for safer alternatives to non viral gene delivery has led to the development of liposomes, cationic polyplexes, microparticles and nanoparticles. Although these alternative vectors have shown promise, degradable nanoparticles are the only non-viral vectors that can provide a targeted intracellular delivery with controlled release properties. Furthermore, the potential advantage of degradable nanoparticles over their non-degradable counterparts is the reduced toxicity and the avoidance of accumulation within the target tissue after repeated administration [104].

Pinteala *et al*. utilized fullerene C_60_ based nanoparticles, coated with hyperbranched polyethylenimine, for gene delivery. Polyethylenimine was extensively investigated as a non-viral vector system due to its high content in amino groups. These provide a great ability to complex and condense with negatively charged DNA or RNA. Fullerene (C_60_), which also showed a good potential in drug delivery, was derivatized onto the surface with hyperbranched polyethylenimine. The interaction between fullerene and polyethylenimine was conveniently followed by UV-Vis spectroscopy: the characteristic peak of C_60_, at λ = 330 nm, decreased during the reaction until disappearance. Depending on the C_60_: polyethylenimine ratios; nanoparticles of 3–80 nm diameters, were obtained in transmission electron microscope (TEM) results. Agarose gel electrophoresis assay showed that C_60_ polyethylenimine had a good DNA binding ability. Intracellular transport is one of the key problems in gene therapy. The lipophilic nature of biological membranes is the major checkpoint to the direct intracellular delivery of potential drugs and molecular probes. SWNTs are the materials of interest as carriers of biologically active molecules, such as small interfering RNAs (siRNAs), due to their ability to cross the cell membranes. The chemical functionalization of SWNTs with hexamethylenediamine (HMDA) and poly (diallyldimethylammonium) chloride (PDDA) has been performed by Krajcik *et al*., to obtain a material which can bind to negatively charged siRNA by electrostatic interactions. PDDA–HMDA–SWNTs did not showed any significant cytotoxic effects on isolated rat heart cells at concentrations up to 10 mg/L. PDDA–HMDA–SWNTs loaded with extracellular signal-regulated kinase (ERK) siRNA were able to cross the cell membrane and suppressed 75% expression of the ERK target proteins in primary cardiomyocytes. PDDA-functionalized SWNTs have been used as an effective carrier system for applications in siRNA-mediated gene silencing [105].

**Table 3:** Delivery of biomolecules through carbon nanotropes [63].

Delivery system	Biological system employed	Results
Antisense oligodeoxynucleotides (ASODNs)-PEI-MWCNT [106]	HeLa cells	ASODN interacted with positively charged amine groups on PEI-MWCNT
Plasmid DNA-carboxylic *f*-MWCNT with embedded Ni [26]	Bal17 B-lymphoma, *ex vivo* B cells and primary neurons	DNA-MWCNT entered in Bal17 B-lymphoma, *ex- vivo* B cells and primary neurons driven by magnetic field and remained highly viable even after transduction
Green fluorescent protein gene-Amino/carboxyl/hydroxyl/alkyl-MWCNT [107]	Human umbilical vein endothelial cells (HUVEC)	Only amino group functionalized MWCNT effectively delivered the pEGFPN1 plasmid into cells
Protective B cell epitope and 1,3-dipolar cycloaddition on SWCNT [108]	BHK 21 cells	B cell epitope was recognized by specific antibodies after being conjugated to SWCNT; mono-peptide-SWCNT led to higher virus neutralizing antibody titers than bis-peptide-SWCNT
Anti-HER2 IgY antibody-SWCNT-CONH_2_ [109]	SK-BR-3 and MCF-7 cells	CNT-antibody complex could detect and selectively kill SK-BR-3 (cancer cells expressing HER2) *in- vitro* in the presence of MCF-7 (non-HER2 expressing) cells
CpG-Oligodeoxynucleotidesand 1,3-dipolar cycloaddition on SWCNT [110]	N.M.	*f*-SWCNT enhanced immunostimulatory properties of ODN CpG; Concentration of IL-6 (stimulated by ODN CpG combined with*f*-SWCNT) in splenocyte cultures decreased more
NF-κB decoy-SWCNT [111]	HeLa cells	Covalent binding of NF-κB decoy on SWCNT greatly reduced the NF-κB dependent gene expression
Oligodeoxynucleotides-SWCNT with maleimide terminal group [112]	N.M.	Hybridization of complementary DNA was highly specific and reversible
DNA-PEI-MWCNT [113]	293cells, COS7 and HepG2 cells	PEI served as anchor point for DNA immobilization; PEI-g-MWCNT exhibited good transfection efficiency for the delivery of DNA
EPO-PEG-8 caprylic/capric glycerides-CNT [114]	Male Wistar rats	Short CNT released twice the amount of EPO than long CNT in rat serum
GnRH-carboxylic-MWCNT [115]	DU 145 cells	GnRH–MWCNT killed Hela cells after internalization by GnRH receptor-positive cells
Single stranded DNA (ssDNA)-SWCNT dotted with Au nanocrystals (Au-SWCNT) [116]	N.M.	Target DNA hybridization to ssDNA probes, which were immobilized on Au-SWCNT
ssDNA-pristine SWCNT [117]	N.M.	ssDNA bound to SWCNT got released by desorption potential
Plasmid DNAand 1,3-dipolar cycloaddition on SWCNT/MWCNT [99]	HeLa cells	*f*-SWCNT complexed with plasmid DNA facilitated higher DNA uptake and gene expression *in vitro*
ssDNA-pristine SWCNT [117]	N.M.	ssDNA bound to SWCNT got released by desorption potential
Plasmid DNAand 1,3-dipolar cycloaddition on SWCNT/MWCNT [99]	HeLa cells	*f*-SWCNT complexed with plasmid DNA facilitated higher DNA uptake and gene expression *in vitro*
siRNA-PEI/pyridinium-*f*-MWCNT [118]	Human lung cancer cell line H1299	Both types of *f*-MWCNTs showed 10%–30% silencing activity and 10%–60% cytotoxicity
siRNA-PDDA-HMDA-SWCNT [105]	Isolated rat heart cells	PDDA-HMDA-SWCNT bound negatively charged siRNA by electrostatic interactions
siRNA-PL-PEG-SWCNT [119]	Human T cells and primary cells	CNT were capable of siRNA delivery to human T cells and PBMCs, and caused RNAi of CXCR4 and CD4 receptors
siRNA/DNA-PL-PEG-SWCNT [120]	HeLa cells	Amine or maleimide terminal of PL-PEG-SWCNT could bind to various biomolecules
TERT siRNA-SWCNT–CONH–(CH_2_)_6−_NH_3_^+^Cl^−^[121]	HeLa cells	TERT siRNA specifically targeted TERT expression and led to growth arrest of tumor cells
Ferritin/SA/biotinyl-3,6-dioxaoctanediamine-1-Pyrenebutanoic acid, succinimidyl ester-SWCNT [122]	N.M.	Pyrenyl groups bound to CNT through strong *π*–*π* interaction, while succinimidyl ester groups worked as anchors for combining proteins
Bovine serum albumin (BSA)-SWCNT-CONH_2_ BSA-MWCNT-CONH_2_[102]	N.M.	90% BSA retained activity after the formation of BSA-CNT conjugates
BSA/SA/Protein A/cytochrome*c*(cyt-*c*)-carboxyl-SWCNT [123]	HL60, Jurkat, HeLa and NIH-3T3 cells	High level of cellular uptake of proteins (molecular weight <80 KDa); cyt-*c*SWCNT conjugate led to higher level of apoptosis in the presence of chloroquine
SA-Biotin-SWCNT [100]	HL60 and Jurkat cells	SA entered cells after binding to SWCNT-biotin transporter
Protein C1q/serum/plasma proteins-pristine SWCNT [124]	Red blood cells	CNT activated human complement through both classical and alternative pathways; C1q bound directly to CNT; fibrinogen and apolipoproteins (AI, AIV and CIII) bound selectively to DWCNT
GRGDSP peptide sequence/IKVAV peptide sequenceand 1,3-dipolar cycloaddition on MWCNT [125]	Jurkat cells, primary splenocytes and neurons	MWCNT exhibited biocompatibility with different cell types; they did not seem to change the neuronal morphology, viability, and basic functions
KGYYG sequence/ GSGVRGDFGSLAPRVARQL sequence and 1,3-dipolar cycloaddition on SWCNT [126]	N.M.	Bound peptides were recognized by monoclonal and polyclonal antibodies; peptide-SWCNT caused immune response
K(FITC)QRMHLRQYELLC sequenceand 1,3-dipolar cycloaddition on SWCNT [99]	3T3 and 3T6 cells	CNT conjugate crossed the cell membrane; FITC-CNTs accumulated mainly in cytoplasm; Peptide-CNT accumulated in nucleus
BV2 microglia/GL261 glioma-pluronic F108-MWCNT [127]	BV2 microglia and GL261 glioma cells	CNT did not lead to proliferative or cytokine changes *in vitro*; they carried DNA and siRNA, and were internalized at higher levels in phagocytic cells than in tumor cells

Note: N.M., not mentioned.

CNTs have a high potential of being used as drug carrierr and biosensors. However, its binding behavior with proteins needs to be studied before the entire potential of CNTs in biological studies can be realized. The affinity of functionalized CNTs to different proteins has been characterized by several studies. Hemoglobin binds with non-functionalized CNTs, and this was identified by Raman spectrum, as this binding does not change Raman luminescence with specific excitation and emission wavelengths (excitation: 514 nm; emission: 520–600 nm). The instant application of these findings is to use non-functionalized CNT as a biosensor to measure H_2_S in blood in which hemoglobin takes about 37% of the total blood volume. Methemoglobin can also bind to MWCNTs which are not functionalized. Knowledge of methemoglobin binding to non-functionalized CNTs is important for its use as a biosensor to measure H_2_S in blood and as a carrier of hemoglobin. Other potential uses, of non-functionalized CNTs are to bind selective groups of proteins [128].

### 5.8. Delivery of Other Drugs

Typically, drug delivery system (DDS) based on the CNTs, had been designed to combat cancers. There are also some promising statements that utilized CNTs as foremost carrier or as adjunct material for the delivery of non-anticancer drugs. Wong *et al.* demonstrated usefulness of the CNT-based DDS in the delivery of small molecule drugs with particular interest to the recent progress of *in vitro* and *in vivo* researches. [129] Apart from anticancer drugs, CNT-based DDS have also been employed for the delivery of other drugs which have been presented in Table 4.

**Table 4:** Delivery of other drugs through carbon nanotropes [63].

Drug delivery system	Dosage and Biological system employed	Drug effect	Effect of drug-CNT conjugate
Theophylline-AL/CNT microsphere [130]	20% (wt%) theophylline per drug-CNT complex	N.M.	More efficient
Dopsone-*O*-(7-azabenzotriazol-1-yl)-*N*,*N*,*N′*,*N′*-tetramethyluronium hexafluorophosphate/*N*,*N*-diisopropylethylamine-*f*-MWCNT [92]	50 μg dopsone per mL of*f*-MWCNT (rat peritoneal macrophages)	Anti-microbial and anti-inflammatory	More efficient
(d-α-Tocopheryl polyethylene glycol 1000 succinate–MWCNT [131]	2.5 μM TPGS (N.M.)	Vitamin E delivery	N.M.
Polyethylene oxide-pentaerythritol triacrylate-[(S)-(+)-ketoprofen]-MWCNT [97]	N.M. (Mouse membrane)	Anti-inflammatory	More efficient
Dxamethasone-CHI–SWCNT [96]	0.5 mg per mL CHI (N.M.)	Anti-inflammatory	More efficient
Amphotericin-B-fluorescein-MWCNT [94]	40 μg mL^−1^ AmB-CNT (Human Jurkat lymphoma T cells)	Antibiotic	More efficient
Carvedilol–MWCNT [132]	20%–60% (wt%) CAR per drug-CNT complex	Anti-hypertensive	More efficient
Acetyl choline-SWCNT [38]	20-50 mg kg^−1^ Ach-CNT (Ach: 4–10 mg kg^−1^)	Alzheimer’s disease therapy agent	More efficient

Note: N.M., not mentioned.

## 6. Challenges and Limitations with Carbon Nanomaterials

The major challenges and limitations associated with the design and characterization of carbon nanomaterials are many including cellular toxicity, optimal drug loading, controlled drug release, delivery and prediction of exact pharmacokinetic profile. There are basic physical issues with matter at such a small scale. Since matter behaves differently on the nano level than it does at micro and macro levels, most of the science at the nanoscale has been devoted to basic research, designed to expand understanding of how matter behaves on this scale. Because nanomaterials have large surface areas relative to their volumes, phenomena such as friction are more critical than they are in larger systems. The small size of nanoparticles may result in significant delay or speed-up in their intended actions. They may accumulate at unintended sites in the body or may provoke unexpected immune system reactions. Cells may adapt to the nanoparticles, modifying the body’s behavior in unforeseen ways. The efficacy of nanoparticles may be adversely affected by their interaction with the cellular environment. For instance, the reticuloendothelial system (RES) may clear nanoscale devices, even “stealth” versions, too rapidly for them to be effective because of the tendency of the RES to phagocytose nanoparticles [133]. Nanoparticles can be taken up by dendritic cells [134] and by macrophages [135]. RES accumulation of nanoparticles could potentially lead to a compromise of the immune system. On the other hand, larger nanoparticles may accumulate in larger organs, leading to toxicity. Perhaps the biggest issue of all is that the physically compromised tumor vasculature may prevent most of the nanodevices from reaching the target cells by vascular transport or diffusion. Alterations in the tumor vasculature may adversely affect the convection of the nanodevices in the blood stream [136]. Local cell density and other stromal features may hamper drug or nanodevice diffusion through tumoral tissue.

## 7. Recent Patents on Carbon Nanotropes in Drug Delivery

This section accentuates the patents granted at USPTO for the advancements and expediency of the carbon nanomaterials based research in the field of drug delivery. The invention by Scheinberg *et al.* provide soluble SWNT constructs functionalized with a plurality of a targeting moiety and one or more payload molecules attached thereto. These molecules were attached with a DNA or other oligomer platform put together with SWNTs. These constructs involved a radionuclide or contrast agent and were effective as diagnostic and therapeutic agents as such. Methods presented in this invention were used to diagnose or locate a cancer, treating a cancer, bring forth an immune response against a cancer or to deliver an anticancer drug *in situ* via an enzymatic nanofactory, using soluble SWNT constructs [137].

Wilson *et al.* described a novel method which encompassed the use of water-soluble cationic fullerene derivatives to improve the plant genetic transformation. Cationic fullerene derivatives accomplished the DNA binding and compaction activity and provide with a new method to deliver DNA into plant cells for transformation. However, water-soluble fullerene derivatives with anionic or non polar substituents showed antioxidant (free radical scavenging) activity and offered improved yields and efficiency of plant transformation methods, such as biolistic or electroporation methods. This method limits cellular damage and cell death which could result into higher yields of viable transformed cells [138].

Compositions and methods for administering a therapeutic agent to a mammal are disclosed in this invention. The compositions comprise either (i) vesicles comprising of an amphiphilic substituted fullerene, wherein the therapeutic agent is present in the vesicle interior or between layers of the vesicle wall; (ii) a substituted fullerene, comprising a fullerene core and a functional moiety, wherein the therapeutic agent is associated with the substituted fullerene; or (iii) carbon nanotubes, wherein the therapeutic agent is covalently bonded to the carbon nanotubes [139].

A functionalized single wall carbon nanotube (SWCNT) complexed with nanochitosan, for use in the delivery of bioaffecting substances and diagnostic applications, has been given in this invention. fSWCNT Complexed with the chitosan NG042 was used for delivery of DNA-encoding EGFP reporter protein and peptide. The results demonstrate that the described CNT-chitosan hybrid nanoparticles exhibit significantly higher transfection efficiency *in vivo* than chitosan alone. Furthermore, the functionalized nanotubes were tested for peptide transfer into HEK293 cells. The results showed that the hybrid nanoparticles efficiently transferred peptides. Together, these results show that hybrid SWCNT-chitosan particles increase DNA and peptide transfer into cells [140].

## 8. Conclusions

Carbon nanomateials have provided an excellent technology platform in the field of drug delivery. Many achievements have been evidenced in delievering drugs such as; anticancer, neurodegenerative disorders, antimycobacterials, antiinflammaroy, topical agents, biomolecules, *etc.* However, there are several limitations associated with the design and characterization of these nanomaterials which include; cellular toxicity, ability to achieve optimal drug loading on the nanotropes, to control drug release, delivery and prediction of exact pharmacokinetic profile. Moreover, carbon nanotropes are considered as a novel paradigm in drug delivery which not only improve the ability to deliver drugs with wide systemic and topical applications but also the ability to control stability, solubility and drug carrying capacity [141]. Thus, it can be concluded that the emergence of nanotherapeutics/nanoformulations provide the pharmaceutical scientists with a tool which, when developed, could revolutionize drug delivery research and the carbon nanotropes promises to be an integral part of it.
